# Effects of *Clostridium butyricum* and a Bacteriophage Cocktail on Growth Performance, Serum Biochemistry, Digestive Enzyme Activities, Intestinal Morphology, Immune Responses, and the Intestinal Microbiota in Rabbits

**DOI:** 10.3390/antibiotics10111347

**Published:** 2021-11-04

**Authors:** Pan Huang, Xuemei Cui, Zhipeng Wang, Chenwen Xiao, Quanan Ji, Qiang Wei, Yee Huang, Guolian Bao, Yan Liu

**Affiliations:** 1 Institute of Animal Husbandry and Veterinary Medicine, Zhejiang Academy of Agricultural Sciences, Hangzhou 310021, China; huangpan202109@163.com (P.H.); maycui@zju.edu.cn (X.C.); zhipengwang688@163.cn (Z.W.); xiaochenwen_xcw@163.com (C.X.); jiquanan@hotmail.com (Q.J.); weiqrabbit@163.com (Q.W.); julieyee2017@outlook.com (Y.H.); 2 College of Chemistry and Life Science, Zhejiang Normal University, Jinhua 321004, China

**Keywords:** antibiotic alternatives, rabbits, growth performance, intestinal morphology, intestinal barrier, caecal microbiota

## Abstract

The objective of this study was to assess the effects of dietary supplementation with *Clostridium butyricum* (CB) and a bacteriophage cocktail (BP) on growth performance, serum biochemical parameters, intestinal digestive and oxidase enzymes, intestinal morphology, immune responses, and the cecum microbiota in rabbits. In total, 108 New Zealand rabbits (5 weeks old) were randomly and equally allotted into three dietary treatment groups (four replicates per treatment, n = 36/treatment): (1) the control (CN) group—rabbits fed the basal diet; (2) CB group—rabbits fed the basal diet supplemented with 100 mg/kg diet *Clostridium butyricum*; and (3) BP group—rabbits fed the basal diet supplemented with 200 mg/kg diet BP cocktail, respectively, for 6 weeks. Compared with the CN diet, dietary CB and BP inclusion increased the average daily gain (ADG) and average daily feed intake (ADFI) and decreased the feed/gain (F/G) ratio of rabbits. Furthermore, CB increased the digestive enzyme activity (α-amylase and trypsin in the ileum); the chymotrypsin activity was also significantly increased in the duodenum and jejunum. Supplementation with CB significantly enhanced antioxidant capacity (SOD and GSH-Px) in the jejunum and ileum and reduced MDA levels. Additionally, rabbits fed CB had significantly elevated villus height (V) and (V/C) ratios but reduced crypt depth (C). Moreover, dietary CB supplementation markedly increased the ileal expression of tight junction proteins (occludin, ZO-1, and claudin-1) and increased secretory immunoglobulin A (sIgA) production. High-throughput sequencing indicated that the microbiota in the rabbit intestine was altered by CB and BP. Venn diagrams and heatmap plots revealed that the gut microbial community composition varied obviously among rabbits fed different diets. Specifically, CB increased the relative abundance of beneficial bacteria to maintain intestinal barrier homeostasis, whereas BP decreased the relative abundance of Gammaproteobacteria, which included a plenty of pathogenic bacteria.

## 1. Introduction

Due to the fragile digestive system of rabbits, they are prone to gastrointestinal diseases [1]. The high incidence of diarrhea and mortality in weaned rabbits present severe problems for rabbit breeding. Rabbit diarrhea is mainly caused by pathogens such as *Escherichia coli*, *Clostridium perfringens*, and *Salmonella*, and some external stresses influence gut health, including feed replacement, weanling stress, and climate change, which result in heavy economic losses in the rabbit production industry [2]. Antibiotics have played an important role in controlling diseases and enhancing growth in animals [3]. However, the antibiotic resistance crisis is becoming incredibly difficult due to the indiscriminate employment of antibiotics, which poses a significant threat to animals and public health. Antibiotic treatment can improve the tolerance of pathogenic bacteria and destroy the normal intestinal microbiota [4]. For these reasons, the discovery and development of new antibiotic alternatives (such as traditional Chinese medicine and probiotics) have attracted increasing attention from researchers. Probiotics such as yeast *Sacharomyces cerevisiae* have been evaluated among others as potential feed supplements to improve animal performance, improve feed utilization, enhance feed digestibility, and reduce the number of pathogens [5]. Supplementation of the rabbit’s diet with lysozyme (LZM) was able to improve the growth performance and hematological and serum biochemical parameters [6]. In addition, ginger powder and Chinese herbal medicine supplementation have been reported to improve the production performance, serum metabolites, and antioxidant activity of layer hens [7].

Currently, with the restrictions of antibiotic use, it is urgent to find novel alternatives to replace antibiotics that could positively influence gut health by improving immune responses and the barrier function. The development of modern animal husbandry tends to produce green and safe animal products. Probiotics and beneficial bacteria have been used widely and are considered as potential alternatives to antibiotics, representing a novel nonantibiotic approach to modulate gut function [8]. *Clostridium butyricum* (CB) is a butyric acid-producing Gram-positive anaerobic bacterium that has been isolated from the intestines of healthy humans and animals [9]. Studies have suggested that CB administration improves animal growth, enhances immune function, improves intestinal microecology balance, decreases *Escherichia coli* colonization and infection, and reduces intestinal barrier damage and permeability [10,11]. Bacteriophages (BPs) can specifically lyse bacteria; are harmless, safe, and efficient; and can be developed as ecofriendly antibacterial agents. Phages infect only specific hosts according to the specificity of the receptor (such as proteins, lipopolysaccharides, teichoic acids, and flagella) present on the surface of the host bacteria [12]. Moreover, phages are used as a substitute for antibiotics for many antibiotic-resistant bacterial strains and as biocontrol agents in the agriculture and aquaculture industries [13]. The main advantage of bacteriophages is their specificity, and treatments can be designed to specifically target pathogenic bacteria while not negatively affecting the normal microbiota, which can compete successfully with other potential pathogens [14].

Therefore, the present study aims to (i) investigate the influence of CB and a BP cocktail on growth performance, intestinal digestive and antioxidant activity, intestinal morphology, and intestinal immune barrier function in growing rabbits and (ii) characterize the effect of CB and a BP cocktail on the cecum bacterial community of rabbits.

## 2. Results

### 2.1. Growth Performance

The effects of dietary CB and BP supplementation on the growth performance of rabbits are presented in Table 1. The rabbits fed diets with CB and BP showed significantly higher ADG (*p* < 0.05) than those fed a basal diet (CN). Moreover, supplementation with CB resulted in a significant increase in ADFI (*p* < 0.05) compared with that of the CN diet. The F/G ratio in the groups supplemented with CB and BP was significantly lower (*p* < 0.05) than that in the nonsupplemented group. These results indicated that CB and BP significantly improved the growth performance of rabbits.

### 2.2. Serum Biochemical Index Analysis

**Figure 1:**
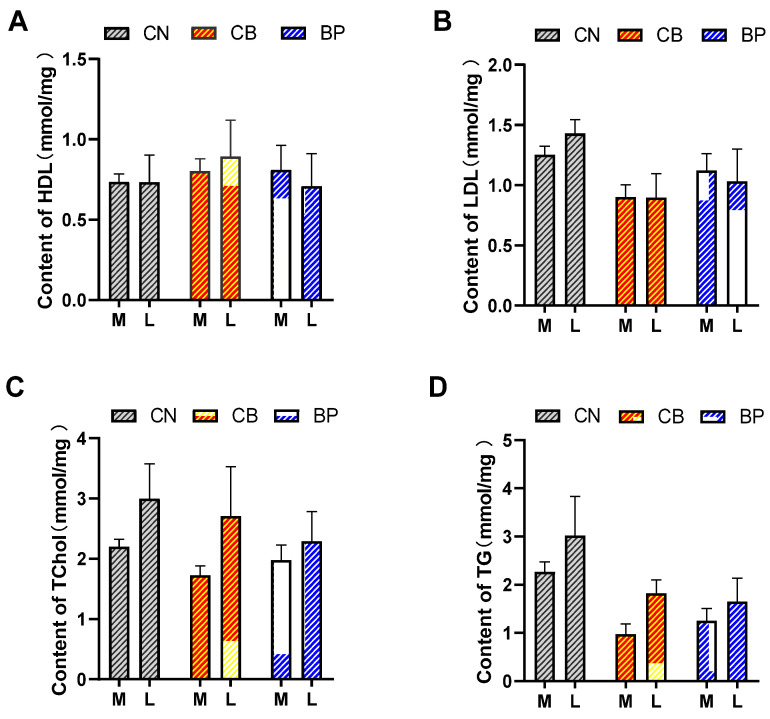
Effects of CN, CB, and BP on serum parameters in rabbits. (**A**–**D**) Serum concentrations of HDL, LDL, Tchol, and TG at the middle phase (M = 21 days) and later phase (L = 42 days). CN, rabbits were fed a basal diet; CB, rabbits were fed a basal diet supplemented with *Clostridium butyricum*; BP, rabbits were fed a basal diet supplemented with a bacteriophage cocktail. HDL, high-density lipoprotein; LDL, low density; Tchol, total cholesterol; TG, triglyceride; (values are expressed as the mean ± SE, n = 8).

The effects of CB and BP on serum HDL, LDL, Tchol, and TG in rabbits are shown in Figure 1A–D. Adding CB and BP to the basic diet had no significant effect on the serum biochemical indexes of rabbits compared to those of the CN group rabbits. Rabbits in the CB and BP groups had obviously decreased LDL and TG concentrations compared with those of the rabbits in the CN group at days 21 and 42.

### 2.3. Digestive Enzyme Activity

**Figure 2:**
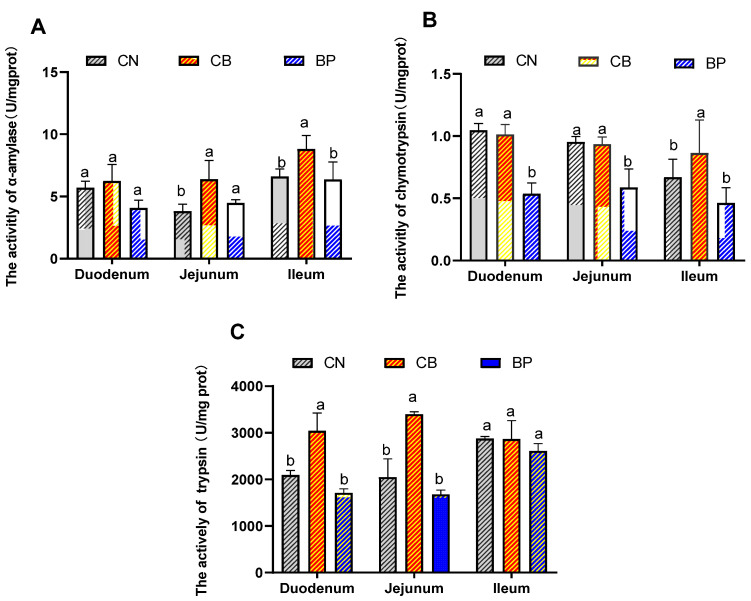
Effects of CN, CB, and BP on digestive enzyme activity in weaning rabbits. (**A**–**C**) α-amylase, chymotrypsin, and trypsin activity in small intestinal contents. CN, rabbits were fed a basal diet; CB, rabbits were fed a basal diet supplemented with *Clostridium butyricum*; BP, rabbits were fed a basal diet supplemented with a bacteriophage cocktail (values are expressed as the mean ± SE, n = 8; ^a,b^ Mean values within a row with unlike superscript letters were significantly different (*p* < 0.05).

The effects of CB and BP on the activities of digestive enzymes in the small intestine of rabbits are shown in Figure 2A–C. The α-amylase activity in the jejunum and ileum of the CB group was significantly increased (*p* < 0.05) compared with that of the CN group. The chymotrypsin activity of the CB group was also significantly increased (*p* < 0.05) in the ileum. However, the chymotrypsin activity in the duodenum and jejunum was slightly decreased in the BP group. Significantly elevated trypsin activity in the duodenum and jejunum was detected (*p* < 0.05) in the CB group compared to that of the CN group.

### 2.4. Antioxidant Status of Small Intestinal Tissues

**Figure 3:**
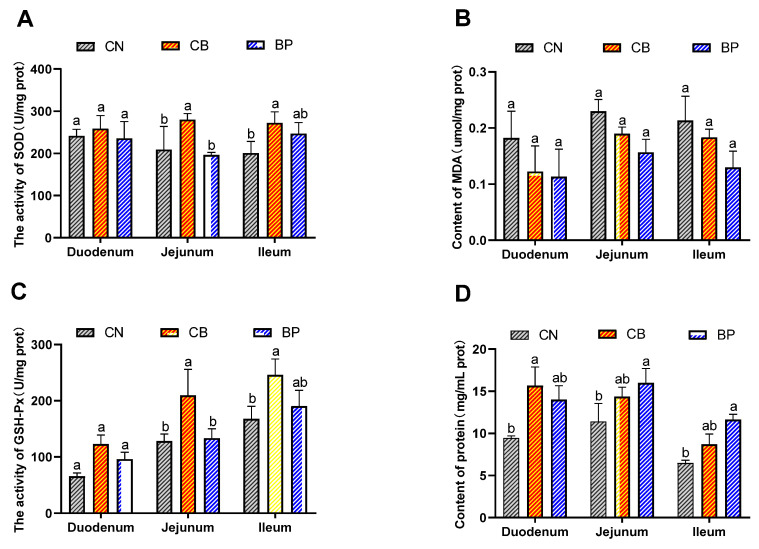
Effects of CN, CB, and BP on antioxidant enzyme activity in weaning rabbits. (**A**–**D**) SOD and GSH-Px activity, protein activity and MDA content in small intestinal tissues. CN, rabbits were fed a basal diet; CB, rabbits were fed a basal diet supplemented with *Clostridium butyricum*; BP, rabbits were fed a basal diet supplemented with a bacteriophage cocktail (values are expressed as the mean ± SE, n = 8; ^a,b^ Mean values within a row with unlike superscript letters were significantly different (*p* < 0.05).

The antioxidant status of the small intestinal tissues of rabbits is shown in Figure 3A–D. SOD and GSH-Px activities and protein content were found to be improved in all rabbits fed CB compared to those of rabbits in the CN group. Supplementation with CB significantly increased the GSH-Px (*p* < 0.05) and SOD activity (*p* < 0.05) in the jejunum and ileum. However, the BP group showed no significant differences in SOD or GSH-Px activity in the small intestine. The two treatment groups displayed a trend toward a reduced MDA concentration in the small intestine. The protein concentration in the duodenum and ileum of the CB group was significantly increased (*p* < 0.01). Unexpectedly, the protein concentration in the jejunum and ileum of the BP group was significantly increased (*p* < 0.05) compared to that of the CN group.

### 2.5. Morphological Assessment of the Small Intestinal Mucosa

**Figure 4:**
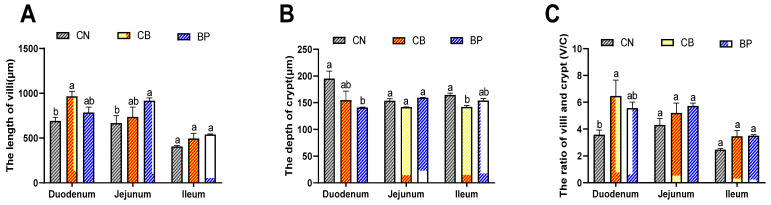

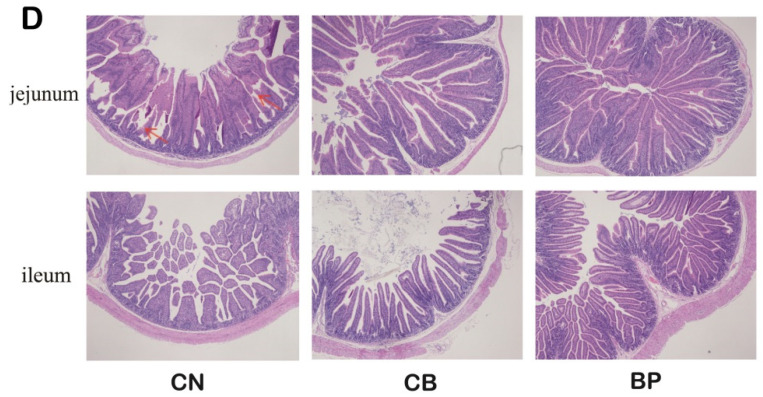
Effects of CN, CB, and BP on the morphology of the small intestinal mucosa in rabbits. (**A**–**C**) Villus length and crypt depth in the small intestinal mucosa, as well as the ratio of villus length to crypt depth. (**D**) H&E-stained section images (40×) of the jejunum and ileum. CN, rabbits were fed a basal diet; CB, rabbits were fed a basal diet supplemented with *Clostridium butyricum*; BP, rabbits were fed a basal diet supplemented with a bacteriophage cocktail (values are expressed as the mean ± SE, n = 8; ^a,b^ Mean values within a row with unlike superscript letters were significantly different (*p* < 0.05).

The morphology of the small intestinal mucosa in rabbits was analyzed, as shown in Figure 4A–C. Compared with the CN group, the CB group displayed obviously elongated villi in the duodenum (*p* < 0.01), and the BP groups showed longer villi in the jejunum (*p* < 0.05). On the other hand, the CB treatments decreased the crypt depth of rabbits. In the duodenum, a clear decrease (*p* < 0.05 or *p* < 0.01) in crypt depth was detected in the CB and BP groups compared with that of the CN group. The crypt depth of the CB groups was markedly reduced ileum compared with that of the CN group (*p* < 0.05). Subsequently, the villus length-to-crypt depth (V/C) ratio was calculated. In the CB group, the V/C ratio in the duodenum was remarkably increased (*p* < 0.01) compared with that of the CN group, and the data showed that compared with that of the CN group, the V/C ratio in the duodenum of BP groups (*p* < 0.05). Thus, CB and BP can protect the integrity of intestinal villi.

### 2.6. Immune Responses

**Figure 5:**
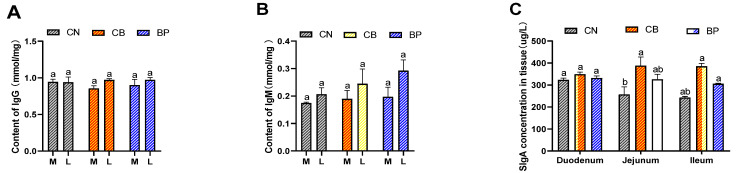
Effects of CN, CB, and BP on IgG and IgM levels and secretory immunoglobulin A in rabbits. (**A**,**B**) Serum concentrations of IgG and IgM at the middle phase (M = 21 days) and later phase (L = 42 days). IgG, immunoglobulin G; IgM, immunoglobulin M; (**C**) secretory immunoglobulin A concentration in tissue. CN, rabbits were fed a basal diet; CB, rabbits were fed a basal diet supplemented with *Clostridium butyricum*; BP, rabbits were fed a basal diet supplemented with a bacteriophage cocktail (values are expressed as the mean ± SE, n = 8; ^a,b^ Mean values within a row with unlike superscript letters were significantly different (*p* < 0.05).

The effect of CB and BP on IgG and IgM levels in serum, and sIgA was analyzed with an ELISA kit (Meimian Industrial, Zhangjiagang, China) according to the manufacturer’s instructions. As shown in Figure 5, CB and BP increased the IgM level in the later stage (42 days) compared to that in the CN group. The sIgA level in the duodenal mucosa was not significantly different among the two treatment groups. The CB treatment group showed a clearly increased (*p* < 0.05) sIgA level in the jejunum compared with that of the CN group. Furthermore, the CB groups displayed clearly increased (*p* < 0.05 or *p* < 0.01) sIgA levels in the ileum compared with that of the CN group.

### 2.7. Tight Junction Protein mRNA Level

**Figure 6:**
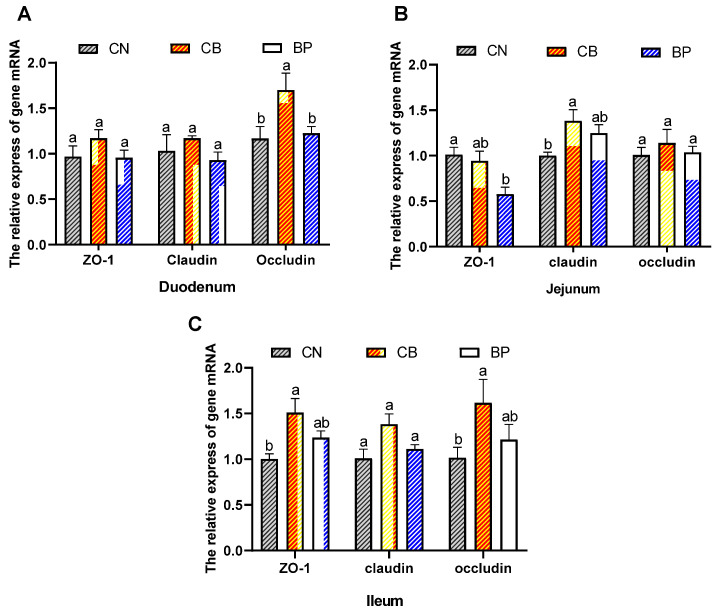
Effects of CB and BP on tight junction protein expression in rabbits. (**A**–**C**) The mRNA levels of tight junction proteins (ZO-1, occludin, claudin) in the duodenum, jejunum, and ileum, respectively. The mRNA level was quantified according to that of the housekeeping gene GAPDH, and the level of gene expression was expressed as the relative value compared to that of the CN group. CN, rabbits were fed a basal diet; CB, rabbits were fed a basal diet supplemented with *Clostridium butyricum*; BP, rabbits were fed a basal diet supplemented with a bacteriophage cocktail (values are expressed as the mean ± SE, n = 8; ^a,b^ Mean values within a row with unlike superscript letters were significantly different (*p* < 0.05).

We evaluated the mRNA expression level of tight junction proteins in rabbits. Figure 6A–C. Shows that in the duodenum, an obvious increase (*p* < 0.05) in occludin mRNA levels was seen in the CB group. In the jejunum, the relative expression of claudin in the CB group was elevated (*p* < 0.05) compared with that in the CN group. In the ileum, the CB group showed clearly upregulated relative mRNA expression of ZO-1 (*p* < 0.05) and occludin (*p* < 0.05) compared with that of the CN group. 

### 2.8. The Effect of Treatment on Cecal Microbial Diversity

**Figure 7:**
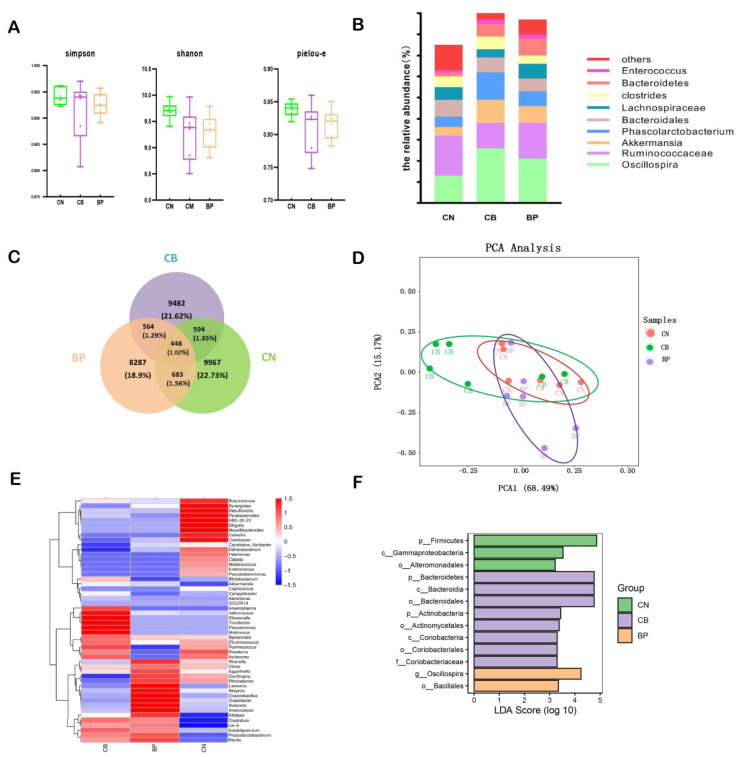
Summary of the microbial community in the cecum contents of rabbits. (**A**) Simpson, Shannon, and Pielou-e indexes of α diversity. (**B**) The relative abundance of the top 20 genera classified as rectal microbiota constituents. (**C**) A Venn diagram summarizing the numbers of common and unique operational taxonomic units (OTUs). (**D**) Nonmetric multidimensional scaling (NMDS) analysis. (**E**) Species composition heat map at the genus level for species clustering. The red color blocks represent genera that were more abundant in that sample than in other samples, and the blue color blocks represent genera that were less abundant in that sample than in other samples. (**F**) Histogram of linear discriminant analysis (LDA) effect values by marker species, from phylum to species in this graph, that were statistically significant (*p* < 0.05) and had an LDA score > 2. CN, rabbits were fed a basal diet; CB, rabbits were fed a basal diet supplemented with *Clostridium butyricum*; BP, rabbits were fed a basal diet supplemented with a bacteriophage cocktail (two samples failed to pass the quality inspection, thus n = 6).

The α diversity of samples from rabbits fed basal diets (CN) or diets supplemented with CB and a BP cocktail was calculated using the Shannon index, Simpson index, and Pielou-e (Figure 7A). There were no differences in the Shannon and Simpson diversity indexes among the experimental groups (*p* > 0.05). At the genus level, *Clostridiales*, *Ruminococcus*, *S24-7*, *Bacteria*, and *Oscillospira* predominated in all groups (Figure 7B). The relative abundance of *Clostridiales* in the CB group was higher than that in the CN group. The cecal microbiota composition is shown in a Venn diagram. A total of 448 OTUs were shared among the three treatment groups (Figure 6C). There was a difference in the microbial community structure of the CB and BP groups compared with that of the CN group (ANOSIM; BP vs. CN: R = 0.02, *p* = 0.3), as indicated by NMDS (Figure 7D) There was a significant difference in the microbiota between the CB (CB vs. CN: R = 0.01, *p* = 0.01) and CN groups. The abundances of these genera for each treatment group were visualized on a heatmap, displaying trends in genus abundance distribution, and the individual treatment groups differed in composition at the genus level (Figure 7E). To further investigate how the composition of fecal bacteria changed, a LEfSe analysis was performed to determine the most differentially abundant genera in all treatment groups. The results showed that some of the biomarkers, from phyla to species, were significantly more abundant in the CN, CB, and BP groups (Figure 7F). Whereas microbiota variety was observed in terms of the *Firmicutes* abundance at the phylum level in the CN group rabbits, the CB group rabbits had significantly greater abundances of *Bacteroidetes* and *Actinobacteria* at the phylum level, and cecal microbiota samples from the BP group had higher relative abundances of *Oscillospira* at the genus level (Figure 7F).

**Figure 8:**
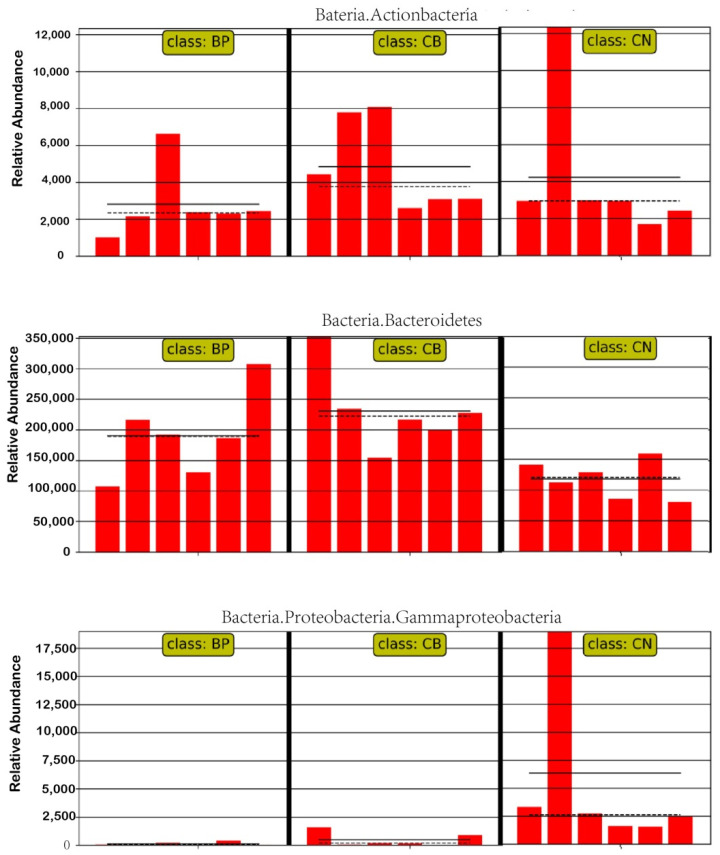
Relative abundance distribution of marker families in different groupings by LEfSe. The mean and median relative abundance values for each taxon in each grouping are identified as solid and dashed lines, respectively. CN, rabbits were fed a basal diet; CB, rabbits were fed a basal diet supplemented with *Clostridium butyricum*; BP, rabbits were fed a basal diet supplemented with a bacteriophage cocktail.

LEfSe analysis was also used to identify the difference between the CN and other treatment groups (Figure 8). We found that the CB group had increased abundances of *Bacteroidetes* and *Actinobacteria*. In addition, the BP cocktail group had a decreased relative abundance of *Gammaproteobacteria*.

## 3. Discussion

*Clostridium butyricum* provides multiple benefits for animals. Published articles indicated that CB administration promotes animal growth and enhances the immune function [15]. Moreover, bacteriophages have received attention due to their natural antimicrobial properties and lower propensity for the development of bacterial resistance [16]. The present study demonstrates the effects of CB and a BP cocktail on the performance and gut health of rabbits. Among these, CB is a known butyrate-producing bacterium and a regulator of gut health [17]. CB exerts its effects through a variety of mechanisms, including stimulating intestinal development and repair, preventing pathogen invasion, and producing antibacterial substances [18]. Takahashi et al. [19] recently reported that the use of CB significantly increased ADG and ADFI in weaned piglets, and Abdel-Latif et al. reported similar findings in broilers [20]. Our data are in agreement with these findings. In this study, the data demonstrated that dietary CB supplementation slightly increased ADG and ADFI. Kim et al. showed that a dietary BP cocktail decreased the F/G ratio and improved growth performance, which is consistent with the results of our feeding experiment [21]. The beneficial effects of BP treatment on growth performance are in agreement with other studies using BPs, which provide support for the use of BPs in rabbit diets. Similarly, Gebru et al. reported that dietary bacteriophage supplementation significantly increased the ADG and F/G ratio of *Salmonella*-challenged pigs [22]. Contrary to our findings, Zeng et al. found that dietary supplementation with 200 mg/kg BP had no beneficial effect on the F/G ratio or the growth performance of pigs; however, dietary supplementation with 400 mg/kg BP increased the final BW, ADG, and ADFI and decreased the F/G ratio [23]. The effects on growth performance could be associated with the dosage, feeding environment, host health status, or even administration level and hygiene status [24]. We measured serum parameters to evaluate nutrition levels in rabbits, including TG, TC, HDLC, and LDLC levels. Our results indicated that dietary CB and BP cocktail supplementation did not affect serum TG, TC, HDL, or LDL levels. Nevertheless, Ghasemi et al. [25] observed that the supplementation of symbiotic reduced circulating cholesterol and LDLC in broilers. 

Digestive enzymes directly influence nutrient digestibility and growth performance. The digestion of fiber is completed under the synergistic action of α-amylase, trypsin, and chymotrypsin, and changes of the activity of any enzyme could affect its decomposition. Three enzymes activities were enhanced to different degrees after supplementation with CB. Protein and starch in mammalian food are mainly digested by trypsin and α-amylase, and a small amount is digested by intestinal bacteria [26]. Previous studies found that probiotics *Bacillus* enhanced the activity of protease, amylase, and cellulose of young and weaning Rex rabbits [27]. Thus, the results of this study indicated that the digestive function was heightened by CB.

Oxidative stress is increasingly being recognized as a factor in the pathogenesis of intestinal pathologies [28]. Therefore, the antioxidant ability of intestinal tissues is important for animal growth. Antioxidant enzymes can protect the body from oxidative damage and enhance defense [29]. Specifically, GSH-Px and SOD remove peroxide and hydroxyl radicals and protect the structure and function of cell membranes [30]. MDA is one of the most frequently used indicators of lipid peroxidation. The inclusion of *Lycium barbarum polysaccharide* (LBP) in the diet has been shown to increase SOD and GSH-Px activities but decrease MDA content in the serum and liver in broilers [31]. CB has also been used to reveal significantly decreased MDA content and enhanced total SOD and GSH-Px activities [32,33]. Moreover, Liao et al. found that supplementation with CB enhanced antioxidant status and decreased MDA content [34]. Similar to these results, the present experiment showed that the CB treatment group exhibited an upward trend in SOD and GSH-Px activity in the ileum. A preliminary study performed by Przerwa et al. suggested that phage T4 inhibited reactive oxygen species (ROS) production by reducing bacterial levels during infection and lysis by the phage but not via direct effects of the phage [35]. However, our results showed that the BP group had no significant differences in SOD or GSH-Px activity in the small intestine. These results suggest that CB have the persistent positive roles in enhancing the antioxidant capacity of rabbits.

Serum immunoglobulins (IgG and IgM) and intestinal immunity secretory IgA (sIgA) levels play an important role as the main indicators of an animal’s health. In the current experiment, adding BP to the basic diet had no significant effect on serum immune indexes of rabbits compared to those of the CN group rabbits. Dietary CB supplementation promoted the humoral immune response, leading to an increase in serum IgM concentrations. However, Chen et al. found that CB dietary significantly increased the serum IgM content of ducks [36], and Yang et al. indicated that chicks fed diets supplemented with CB had a higher IgM concentration [37]. As the largest immune organ, the intestine is a necessary component of the mucosal immune system, and unique immune functions serve as a physical barrier against invading pathogens [38,39]. sIgA is produced by mucosal plasma cells and is the predominant intestinal immunoglobulin [40]. In agreement with our results, Li et al. found that the oral administration of *Lactobacillus delbrueckii* increased the sIgA concentration in the intestinal mucosa of piglets [41]. Our results indicate that supplementation with CB increased sIgA secretion in the small intestine, especially in the ileum. According to our data, the sIgA level in the mucosa was not significantly different among the BP groups. Zend et al. found that diets supplemented with 400 mg/kg bacteriophage enhanced the content of sIgA of weaned piglets [23]. 

Small intestinal morphology is also a vital factor in the maintenance of normal intestinal functions, as well as the digestive and absorptive capacity of the intestine, which is usually evaluated according to the villus height, crypt depth, and V/C ratio [42]. Long et al. reported that the combined use of CB and *Lactobacillus salivarius* could further improve the intestinal villus length in mice [43]. According to Chen et al., CB increased the duodenal, jejunal, and ileal villus height and jejunal V/C ratio in lipopolysaccharide-challenged weaned piglets [44]. We speculated that CB improved the nutrient absorptive capacity by increasing the intestinal villus height and V/C ratio, which may have contributed to the improved ADFI observed for rabbits. Jejunal villus height has been found to increase in pigs that received BPs, but the BPs did not affect other morphological parameters [45]. Similarly, our feeding trial provided evidence that villus height in the jejunum improved in the BP group; however, it had nonuniform alterations in the individual intestinal segments. Many improvements in intestinal morphology occurred, but the full understanding of the effects of BPs on morphology is incomplete. The intestinal barrier is primarily regulated by tight junctions, which consist of a well-organized junctional structure located at the lumenal side of the intestinal epithelium. ZO-1, occludin, and claudin are three important proteins for tight junctions. In the present study, compared to the CN group, the dietary CB supplementation groups showed an upregulated relative mRNA expression of ZO-1 and occludin in the ileum. We speculated that CB could play a role in enhancing intestinal barrier function to prevent antigen entry that induces inflammatory diseases in rabbits.

There are estimated to be a large number of bacterial species inhabiting the rabbit cecum. In rabbits, the bacterial community of cecum is associated with the maintenance gut health and compromised growth performance. In the gastrointestinal tract, the gut microbiota itself can act as a barrier to exterior pathogens by stimulating the host to produce antimicrobial compounds and by competitive exclusion via consumption of nutrient sources and occupation of attachment sites [46]. In the present study, CB effectively increased the relative abundance of *Akkermansia* at the genus level. *Akkermansia*, belonging to the *Verrucomicrobia* phylum, can improve intestinal barrier integrity [47,48]. CB effectively increased intestinal microbial diversity and abundance, maintaining the intestinal microbiota balance. Zhang et al. suggested that CB influenced microbial metabolism by optimizing the microbiota structure [12]. Our work demonstrated that supplementation with CB altered the cecum microbiota structure, with observably distinctive OTUs appearing and markedly different community structures based on NMDS analyses (*p* = 0.01). In the present study, the abundances of *Bacteroidetes* and *Actinobacteria* in the cecum contents of the CB group were greater than those of the control group. Wang et al. proved that the relative abundance of *Actinobacteria* in the CB group was elevated in piglets [49]. *Actinobacteria* and *Bacteroidetes* play a major role in maintaining the gut barrier homeostasis, as they are good at restraining anaerobes and promoting the proliferation of healthy bacteria to improve the breeding environment for the intestinal flora [50]. It has been determined that bacteriophage influences bacterial community structure and could be harnessed to direct the assembly of bacterial communities to promote microbial homeostasis [51]. BPs could change the functions of the microbiome community by the selective elimination of species from the gut microbiome [52]. In the current experiment, BP supplementation to the basal diet increased the relative abundance of *Oscillospira* bacteria at the genus level. Some studies have suggested that BPs with immunoglobulin Ig-like domains in their capsids bind to the mucosal surfaces of animals and are covered with mucin glycoproteins, which provides phage-mediated antibacterial protection of animal mucosal surfaces [53]. Accordingly, in this study, it was found that BPs effectively decreased the relative abundances of *Proteobacteria* and *Gammaproteobacteria*, including many pathogenic bacteria, such as *Escherichia coli*, *Salmonella*, and other well-known species. These pathogens cause life-threatening gastrointestinal diseases. These results might explain the use of BPs resulting in decreased abundances of pathogenic bacteria, improved intestinal microbiota, and other relative downstream effects. This study is important for the implementation of a strategy to reduce or replace the dietary use of antibiotics by CB and BP cocktails or other alternatives in rabbit production and breeding.

## 4. Materials and Methods

### 4.1. Animals, Treatment, and Designation

Rabbits were obtained from the experimental rabbit farm of the Animal Husbandry and Veterinary Research Institute, Zhejiang Academy of Agricultural Sciences (Hangzhou, China). Rabbit cages are metal cages with a length of 40 cm, a width of 60 cm, and a height of 45 cm. They were arranged by a three-layer assembly with free access to water and with an automatic washout device. The rabbit breeding room temperature was 15–25 °C, and humidity was 60–70%. CB (concentration of 1 × 10^10^ colony-forming units (CFUs) per gram CB powder) was purchased from JBH Bio-Tech (Henan, China). Complex BP cocktail grains containing *Escherichia coli* phage (k88, k99, EC1506), *Salmonella typhimurium, Salmonella enteritidis* phage, and *Clostridium perfringens* type A phage were purchased from Qingdao Nuo’anbaite Biotechnology Co., Ltd. (Qingdao, China). The titer of each bacteriophage in the bacteriophage cocktail was 10^8^ plaque forming units (pfu)/g bacteriophage cocktail.

### 4.2. Experimental Design

A total of 108 New Zealand rabbits (5 weeks old) with a similar average body weight (BW) were used. Over the course of 42 days, all rabbits were housed in steel cages under the same conditions, and all rabbits were randomly and equally allotted into four dietary treatment groups (four replicates per treatment, 9 rabbits/replicate, N = 36/treatment): (1) in the control (CN) group, rabbits were fed a basal diet without any supplementation; (2) in the CB group, rabbits were fed a basal diet supplemented with 100 mg/kg diet CB; and (3) in the BP group, rabbits were fed a basal diet mixed with 200 mg/kg diet BP cocktail. The dosage of additives in this study were based on previous studies, and the basal feed was formulated as shown in Table 2. All rabbits involved in the study were kept under similar management and were offered feed and water ad libitum. The animal experiments were approved by the Ethics Committee of the Zhejiang Academy of Agricultural Sciences. The animal studies were conducted following the principles and guidelines of the Zhejiang Farm Animal Welfare Council of China.

### 4.3. Sample Collection

Six weeks later, a total of eight healthy (take 2/replicate) rabbits were randomly selected from each experimental group and sacrificed by cervical dislocation. Blood samples were collected via rabbit ear artery at days 21 and 42. At day 42, two 3 cm-long segments and one 5 cm-long segment were obtained from the duodenum to ileum; one 3 cm-long fresh tissue sample (duodenum, jejunum, and ileum) was washed with normal saline, the middle segments of which were fixed in 4% paraformaldehyde for villus morphology assessment; and the other 3 cm-long intestinal tissue was immediately transferred into liquid nitrogen for analysis of the mRNA expression of intestinal tight junction proteins. The 5 cm-long intestinal tissue segments were used for collecting mucosal samples, and then, cecal contents were squeezed into a sterile tube, and all the collected samples were immediately stored at −80 °C for further analysis.

### 4.4. Growth Performance Evaluation

Over the course of 42 days, the health states of rabbits were recorded, and the rabbits were weighed every week during the trial. The BW of individual rabbits was measured at the beginning (5 weeks old) and end (11 weeks old) of the experiment. The average daily gain (ADG), average daily feed intake (ADFI), and feed-to-gain (F/G) ratio were calculated by recording the feed intake of rabbits for each dietary treatment during the experimental period.

### 4.5. Serum Biochemical and Immune Index Analysis

Blood samples were collected from the rabbits via rabbit ear artery in a 5 mL tube at days 21 and 42. Blood samples were then centrifuged at 3000× *g* for 10 min at 4 °C to recover the serum, and the obtained serum was immediately stored at −20 °C for further analysis. Biochemical parameters, including total cholesterol (Tchol), high-density lipoprotein (HDL), low-density lipoprotein (LDL), and triglyceride (TG) levels, were determined using a GS200 Automatic Biochemical Analyzer (Hangzhou Genius Electronics Co., Ltd., Hangzhou, China). Serum immunoglobulins (IgG and IgM) were evaluated using commercial reagent kits purchased from Shanghai Enzyme-linked Biotechnology Co., Ltd. (Shanghai, China) following the protocol instructions.

### 4.6. Digestive Enzyme Activity and Antioxidant Indexes 

The activities of α-amylase, trypsin, and chymotrypsin in the small intestinal mucosa were determined using commercial kits from Nanjing Jian Cheng Bioengineering Institute (Nanjing, China) according to the manufacturer’s manuals. The protein content was determined according to the instructions of the BCA protein detection kit. Antioxidant indexes, including glutathione peroxidase (GSH-Px), superoxide dismutase (SOD) activities, and malondialdehyde (MDA) content, in small intestinal tissues were measured using reagent kits purchased from Nanjing Jian Cheng Bioengineering Institute (Nanjing, China) following the manufacturer’s instructions.

### 4.7. Morphological Measurement of the Small Intestinal Mucosa 

Small intestinal tissue samples immersed and fixed in 4% paraformaldehyde solution for more than 24 h were cut into small pieces of approximately 1 cm^2^. Subsequently, the sections were dehydrated, embedded, trimmed, sectioned, stained with hematoxylin and eosin, and sealed. The small intestinal villus length (V) and crypt depth (C) were observed by light microscopy and measured by the Image-Pro Plus 6.0 software analysis program (Image-Pro Plus 6.0 Media Cybernetics, Rockville, MD, USA). At least five villi and crypts were randomly selected for each slice, and the measured data were taken as the average. The V/C ratio was calculated.

### 4.8. Secretory Immunoglobulin A

Small intestinal tissue sections were prepared for the analysis of secretory immunoglobulin A (sIgA). Approximately 0.1 g of small intestinal mucosa was accurately weighed, homogenized in saline, and centrifuged at 8000× *g* for 20 min at 4 °C to collect the supernatant. Subsequently, according to the instructions, the level of sIgA in the small intestinal mucosa was quantified by an enzyme-linked immunosorbent assay (ELISA) kit (Mlbio, Shanghai, China).

### 4.9. Quantitative PCR Analysis of Gene Expression

Fifty milligrams of intestinal samples was homogenized in 1 mL lysates using a homogenizer, and the samples were centrifuged at 3000× *g* for 10 min. Then, 450 µL supernatant was added to an RNAiso^®^ Plus Kit (BioTek, Beijing, China) in compliance with the manufacturer’s instructions. The total RNA of the sample was extracted using a fully automated nucleic acid extractor (Auto-Pure 32SH, Allsheng, Hangzhou, China). RNA integrity and purity were assessed by 1.0% agarose gel electrophoresis and a NanoDrop 2000 (Thermo Scientific, Waltham, USA). The generated RNA was stored at −80 °C for subsequent study. Quantitative PCR (Q-PCR) was performed to estimate the gene expression of zonula occludens-1 (ZO-1), claudin, and occludin using a *TransScript*^®^ II Green One-Step qRT-PCR SuperMix Kit (TRAN, Beijing, China) and an ABI Prism 7500 Connect™ Real-Time PCR Detection System (Bio-Rad, Hercules, CA, USA). All the detected genes and their primers are listed in Table 3. The reaction mixture (20 μL) contained 10 μL of 2 × *TransStart*^®^ Tip Green qPCR SuperMix, 0.4 μL of *TransStart*^®^ II One-step RT Enzyme Mix, 0.4 μL of Passive Reference Dye, 0.4 μL of each primer, 1.6 μL of template RNA, and 6.8 μL of RNase-free water. The PCR cycling conditions were set as follows: initial denaturation at 50 °C for 5 min and 94 °C for 30 s, followed by 40 cycles of amplification at 94 °C for 5 s and 60 °C for 34 s. All reactions were run in triplicate. After the reactions were completed, the purity of the PCR products was confirmed by melting curve analysis. The relative expression of gene mRNA levels was analyzed by using the 2^−ΔΔCt^ method [54]; glyceraldehyde-3-phosphate dehydrogenase (GAPDH) was used as an inner reference gen, and the control group was used as the calibrator group for the duodenum, jejunum, and ileum.

### 4.10. High-Throughput Sequencing of the Cecum Microbiota

Total genomic DNA was extracted from cecum content samples using a MoBio Power Soil DNA Isolation Kit (Mo Bio Laboratories, Carlsbad, CA, USA) according to the manufacturer’s protocol. After purification, the DNA sample was diluted to 1 ng/µL using sterile water. In brief, the V3-V4 region of the standard bacterial 16S rRNA gene was amplified using specific primers (F: 5′-ACTCCTACGGGAGGCAGCA-3′ and R: 5′-GGACTACHVGGGTWTCTAAT-3′). The PCR products were purified using a GeneJET Gel Extraction Kit (Thermo Scientific, USA). Libraries were prepared using a TruSeq Nano DNA LT Library Prep Kit for Illumina (New England Biolabs, USA). Related sequencing was performed using the Illumina MiSeq platform (Illumina Technologies, San Diego, CA, USA). All clean reads were clustered into operational taxonomic units (OTUs) at the 97% similarity level, and representative sequences were annotated using a Naive Bayes classifier (version 2.2) in QIIME2 software. The gut microbial diversity was evaluated by α diversity (Shannon index, Simpson index, and Pielou-e). A Venn diagram summarizing the numbers of common and unique OTUs was produced. Analysis of similarities (ANOSIM) was performed to analyze the dissimilarity in beta diversity of the bacterial communities among treatment groups by using mothur (version 1.39.5). Principal component analysis (PCA) was used to assess the clustering of cecum microbial samples. Linear discriminant analysis (LDA) with effect size (LEfSe) was used to analyze the phylum-to-genus composition of the colonic microbial community, for which the LDA score threshold was set to greater than 2. 

### 4.11. Statistical Analysis

All the experimental data analyses were performed using one-way analysis of variance (ANOVA) and Duncan’s multiple comparison tests using the SPSS 19.0 statistical package (SPSS Inc., Chicago, IL, USA). All data are expressed as the mean ± standard deviation (M ± SE). The results with a *p* value less than 0.05 or 0.01 were considered statistically significant. The figures were generated by using GraphPad Prism 8.0 (GraphPad Software Inc., La Jolla, CA, USA).

## 5. Conclusions

Adding CB and BP to the basic diet had no significant effect on the serum biochemical indexes. CB supplementation improves the growth performance, elevates the digestive enzymatic activity, enhances the morphology and development of the intestinal mucosa, improves antioxidant capacity, and maintains mucosal barrier integrity. BP supplementation improves the growth performance and decreases the relative abundance of pathogenic bacteria to improve growth performance. In conclusion, the maximum improvement was recorded with CB supplementation.

## Figures and Tables

**Table 1:** Effects of dietary CN, CB, or BP supplementation on the growth performance of rabbits.

Parameter	CN	CB	BP	*p*-Value
(5 weeks old) Initial BW (g)	682.8 ± 10.03	690.4 ± 52.17	692.1 ± 45.72	0.942
(11 weeks old) Final BW (g)	1215 ± 30.38 ^b^	1354 ± 41.17.1 ^a^	1351 ± 34.84 ^a^	0.0179
ADG (g)	12.62 ± 1.41 ^b^	15.80 ± 2.05 ^a^	15.68 ± 1.28 ^a^	0.0273
ADFI (g)	105.24 ± 3.24 ^b^	109.83 ± 3.32 ^a^	108.73 ± 3.62 ^ab^	0.0484
F/G	8.41 ± 1.61 ^b^	6.95 ± 1.93 ^a^	6.93 ± 1.06 ^a^	0.0379

ADG, average daily body weight gain; ADFI, average daily feed intake; F/G (ADFI/ADG), the ratio of feed intake to body weight gain, indicating feeding efficiency (42 days). Feed amount was calculated based on dry weight. CN, rabbits were fed a basal diet; CB, rabbits were fed a basal diet supplemented with Clostridium butyricum; BP, rabbits were fed a basal diet supplemented with a bacteriophage cocktail (values are expressed as the mean ± SE, n = 36; ^a,b^ Mean values within a row with unlike superscript letters were significantly different (*p* < 0.05).

**Table 2:** Composition and nutrient levels of the basal diet.

Diet Composition	Percentage (%)	Components	Nutrient Levels
Corn	14	Total energy (kCAL.kg^−1^)	3826.88
Bran	18	Crude protein (%)	13.42
Bean cake	8	Calcium (%)	1.43
Grass powder	30.67	Total phosphorus (%)	0.51
Malt root	10	Lysine (%)	0.719
Big bran	16	Methionine (%)	0.234
Mountain flour	0.83	Cystine (%)	0.268
Salt	0.5		
Premix ^a^	2		

^a^ Supplied the following per kg of diet: iron, 50 mg; copper, 10 mg; manganese, 5 mg; zinc, 25 mg; iodine, 0.2 mg; selenium, 0.15 mg; cobalt, 0.1 mg; VA, 6000 IU; VD, 1000 IU; VE, 30 mg; VK3, l μg; VB1, 2 mg; VB2, 6 mg; VB3, 50 mg; VB6, 2 mg; folic acid, 5 mg; pantothenic acid, 20 mg; choline (choline chloride), 50 mg; and biotin, 200 μg.

**Table 3:** Primer sequences for genes for Q-PCR.

* Gene	Prime Sequence (5′→3′)	Size (bp)	Accession
ZO-1	F: CCTGCGAAACCCACCAAA	293	XM_008269782.1
	R: ATGCTGTCGAAAGGTCAGGG		
Claudin	F: GCAAGAGGCCGTATCCAGAG	193	NM_001089316.1
	R: AGTCCGTCTCGTAGTGGTCT		
Occludin	F: GGAGCAAAAGATGCGCATGG	207	XM_008262320.1
	R: AATTGACAGGGGTCAAAGGGT		
GAPDH	F: TGTTTGTGATGGGCGTGAA	129	NC_013676.1
	R: CCTCCACAATGCCGAAGT		

* ZO-1, zonula occludens-1; GAPDH glyceraldehyde-3-phosphate dehydrogenase.

## Data Availability

Data are contained within the article.
